# An evaluation of matrix metalloproteinase-9 (Mmp-9) and tissue inhibitor metalloproteinase-1 (Timp-1) Serum levels and the Mmp-9/Timp-1 Ratio in Covid-19 patients

**DOI:** 10.4314/ahs.v23i1.5

**Published:** 2023-03

**Authors:** Nazlim Aktug Demir, Sema Yilmaz Kirik, Sua Sumer, Onur Ural, Hatice Esranur Kiratlı, Husamettin Vatansev, Esra Paydas Hayatsal, Ugur Arslan, Hakan Cebeci, Lutfi Saltuk Demir

**Affiliations:** 1 Selcuk University, Faculty of Medicine, Department of Infectious Diseases and Clinical Microbiology 42250 Konya, Turkey; 2 Selcuk University, Faculty of Medicine, Department of Medical Biochemistry 42250 Konya, Turkey; 3 Selcuk University, Faculty of Medicine, Department of Medical Microbiology 42250 Konya, Turkey; 4 Selcuk University, Faculty of Medicine, Department of Radiology 42250 Konya, Turkey; 5 Necmettin Erbakan University, Faculty of Medicine, Department of Public Health 42080 Konya, Turkey

**Keywords:** COVID-19, MMP-9, TIMP-1

## Abstract

**Background:**

The progression of COVID-19 has different clinical presentations, which raises a number of immunological questions.

**Objectives:**

This study aimed to investigate MMP-9 and TIMP-1 levels in patients diagnosed with COVID-19 and whether the MMP-9/TIMP-1 ratio is associated with lung involvement in COVID-19.

**Methods:**

This study was conducted with 192 patients and 45 healthy controls. ELISA was used to measure the MMP-9 and TIMP-1.

**Results:**

The MMP-9 and TIMP-1 levels of the patients were found to be higher than those of the controls. MMP-9 and TIMP-1 were detected more in patients with lung involvement on chest CT scans than in those with no lung involvement on chest CT scans. A comparison of lung involvement levels revealed no difference was found between the groups. The MMP-9/TIMP-1 ratio was 5.8 in the group with lung involvement on chest CT scans and 6.1 in the group without lung involvement on chest CT scans. No difference was found between the two groups. A comparison with respect to lung involvement levels showed that the MMP-9/TIMP-1 ratio difference was found between the groups.

**Conclusion:**

Diagnostic and treatment methods targeting MMP-9 activity or neutrophil activation may be important in predicting lung involvement in COVID-19 and directing clinical outcomes.

## Introduction

Coronavirus disease 2019 (COVID-19) is an infectious disease caused by SARS-CoV-2 that has led to a global pandemic and severely stressed many health systems worldwide 1. COVID-19 has a large variety of clinical outcomes, from its asymptomatic form to death associated with severe lung damage. The progression of COVID-19 has different clinical presentations, which raises a number of immunological questions.

Matrix metalloproteinases (MMPs) belong to a family of Zn++- and Ca++-dependent endopeptidases that degrade extracellular matrix (ECM) components. Zinc ions are required for MMP activity and a tissue inhibitor metalloproteinase (TIMP) for inhibition. The balance between MMPs and TIMPs plays a fundamental role in preserving cell matrix integrity. When this balance is disturbed, pathologies may occur, including respiratory distress syndrome (RDS) associated with parenchymal destruction, pulmonary fibrosis, bronchiectasis, and asthma 2.

TIMPs, together with cysteine residues in the N-terminal region, bind to the MMP active center that hosts the zinc ion to form MMP-TIMP complexes. This process prevents MMPs from binding to ECM substrates, thus inhibiting MMP activity. In this way, TIMPs play a role in the regulation of ECM synthesis and degradation 3,4. The MMP/TIMP ratio in the lungs regulates ECM synthesis and degradation. Any change in this ratio in the lungs may lead to aggravation of lung injury and lung tissue fibrosis 5.

This study focused on matrix metalloproteinase-9 (MMP-9), a member of the gelatinase group that has type-IV collagenolytic activity and is effective in early alveolar regeneration and subepithelial basal membrane injury 6, and on tissue inhibitor metalloproteinase-1 (TIMP-1), an MMP-9-specific tissue inhibitor 7,8.

This study aimed to investigate MMP-9 and TIMP-1 levels in patients diagnosed with COVID-19 and whether the MMP-9/TIMP-1 ratio is associated with lung involvement in COVID-19.

## Methods

### Patients

This study was conducted with 192 patients who were being monitored and treated for COVID-19 and SARS-CoV-2 RT-PCR positive in XXXXXXX University Medical School Hospital and 45 healthy controls. Patients aged 18 years and older who were not pregnant and had no lung disease other than COVID-19 involvement were included in the study. The patients were first divided into two groups based on the presence of typical lung involvement in COVID-19 on their chest computed tomography (CT) scans. Those who were found to have involvement on their thoracic CT scans were further divided into three groups based on the extent of their lesions. The extent of lung involvement was assessed using chest CT images by a radiologist with 10 years of experience in thorax radiology. There are some semiquantitative indices developed to grade COVID-19 lung involvement with CT. The involvement of lung parenchyma is usually scored for 5 or 4-point scales. We used a novel CT scoring system for pulmonary involvement in COVID-19 and we think this 3-point scoring scale is more practical in clinical use. The groups were:

**Group 1:** Patients who were diagnosed with COVID-19 and had no lung involvement on chest CT scan.

**Group 2:** Patients who were diagnosed with COVID-19 and had lung involvement on chest CT scan.

**2A. Mild involvement:** Patients with 33% or less lung involvement on chest CT scan.

**2B. Moderate involvement:** Patients with 34–66% lung involvement on chest CT scan.

**2C. Severe involvement:** Patients with 67% or more lung involvement on chest CT scan.

The patients' laboratory test results and chest CT scans were retrieved from the hospital's database, and their values for the following factors were recorded: C-reactive protein (CRP), D-dimer, ferritin, lactate dehydrogenase (LDH), international normalized ratio (INR), fibrinogen, alanine aminotransferase (ALT), aspartate aminotransferase (AST), troponin, creatine phosphokinase (CK), procalcitonin (PCT), and SARS-CoV-2 RT-PCR. Serum samples remaining from blood taken at the time of admission for routine tests were stored at -80oC.

### Molecular Studies

At the XXXXX University Medical School Biochemistry Department laboratory, the MMP-9 molecules (Elabscience brand Catalog No. PKSH033431, USA) and TIMP-1 molecules (Elabscience brand Catalog No: E-EL-H0184, USA) were studied.

The sandwich ELISA method was used to measure the MMP-9 and TIMP-1 concentrations in the patients' serum samples. Diluted serum samples were added to a 96-well ELISA plate coated with antibodies specific to these molecules. The plate was then incubated. The subsequent steps were taken separately but followed the same procedure. After aspiration, a biotinylated detection antibody specific to molecular Avidin-Horseradish Peroxidase (HRP) conjugate was added to each micro plate well to be incubated. They were washed first with three repetitions and then five. The substrate solution was added to each well, and a color change was observed. Adding a stop solution ended the enzyme-substrate reaction, and the color was observed to change from blue to yellow. A spectrophotometric measurement of the optical density was carried out at a wavelength of 450±2 nm. Using a concentration graph plotted in line with the standards, the results were assessed for MMP-9 and TIMP-1.

### Ethical Concerns

This work was carried out in accordance with the Declaration of Helsinki (2000) of the World Medical Association. Approval was obtained for the study from the Ethics Committee (E-70632468-050.01.04-4056) and the Republic of Turkey Ministry of Health. This study was supported by the Scientific Research Projects Coordination Unit of XXXXX University (Project number: 20301017).

### Statistical Analysis

The data were analysed for normality using the Kolmogorov Wilk test. The Mann Whitney U test was used to analyse the differences between two groups, the Kruskall Wallis test to analyse differences between three or more groups, and the Mann Whitney U test with the Bonferroni correction as post hoc. Values of p<0.05 were considered statistically significant.

## Results

This study was conducted with 237 subjects: 192 patients and 45 healthy controls. The mean age of the patients was 44 years (18–92) and that of the control group was 38 years (29–57). There were 91 (47.4%) females and 101 (52.6%) males in the patient group, and 21 (46.7%) females and 24 (53.3%) males in the control group. No statistically significant difference was found between the groups in terms of age and gender (p>0.05).

**Figure 1:**
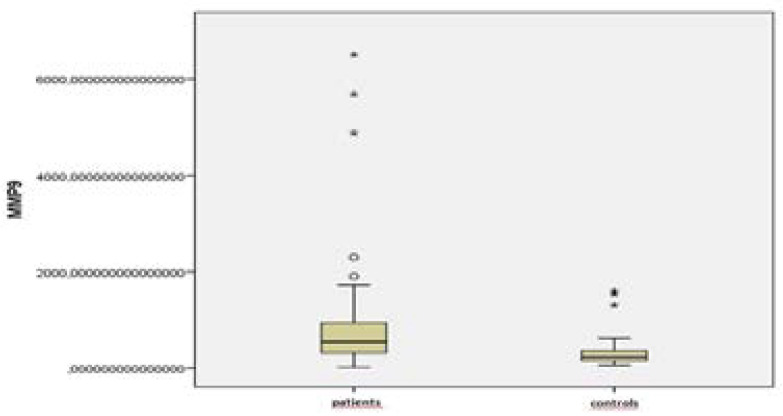
MMP-9 values of the patient and control groups

**Figure 2:**
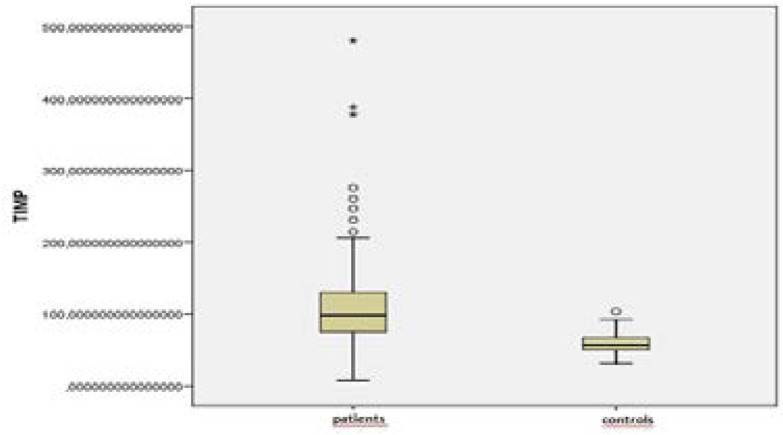
TIMP-1 values of the patient and control groups

The MMP-9 and TIMP-1 levels of the patients were found to be higher than those of the controls (p=0.001, p=0.001) (Figures 1 and 2) (Table 1).

**Table 1:** MMP-9 and TIMP-1 values of the patient and control groups

	Patient (n=192)	Control (n=45)	p
**MMP-9**	550,7 (20,7–6511,9)	242,8 (60,3–1602,4)	**0,001**
**TIMP-1**	98,1 (7,3–480,4)	56,6 (30,8–103,1)	**0,001**

While 143 of th e 192 patients had lung involvement on their chest CT scans (Group 2), 49 patients had no signs of lung involvement (Group 1). The mean age of these 143 patients with lung involvement on their chest CT scans was 49 (18–92) and that of the 49 patients without any signs of lung involvement was 35 (19–90). There were 67 (46.7%) females and 76 (53.3%) males in the patient group having signs of lung involvement, and 24 (49%) females and 25 (51%) males in the group without any signs of lung involvement. While no difference was found between those with and without lung involvement on their chest CT scans with respect to gender (p=0.766), lung involvement appeared to be more common with advancing age (p=0.001).

The CT scans of the 143 patients were then assessed to determine the level of lung involvement. It was found that 110 patients had mild involvement signs (Group 2A), 16 had moderate involvement signs (Group 2B), and 17 had severe involvement signs (Group 2C). When these patient groups were compared with respect to their age and gender, it was found that with increasing age, the level of involvement in the lungs rose.

MMP-9 and TIMP-1 were detected more in patients with lung involvement on chest CT scans than in those with no lung involvement on chest CT scans (p=0.023, p=0.035). A comparison of lung involvement levels revealed no difference was found between the groups (Table 2).

**Table 2:** Patients' MMP-9 and TIMP-1 values and MMP-9/TIMP-1 ratios with respect to lung involvement

	Group 2A (n:110)	Group 2B (n:16)	Group 2C (n:17)	p
**MMP-9**	430,8 (20,7–5685,6)	513,3 (37,6–1907,8)	661,9 (85,9–6511,9)	0,215
**TIMP-1**	97,9 (7,9–387,9)	102,7 (66,7–377,5)	109,3 (7,3–480,4)	0,374
**MMP-9/TIMP-1**	6,0 (0,1–77,1)	5 (0,4–22,1)	4,6 (0,6–235,4)	0,401

The MMP-9/TIMP-1 ratio was 5.8 (0.11–235.49) in the group with lung involvement on chest CT scans and 6.1 (1.14–25.02) in the group without lung involvement on chest CT scans. No difference was found between the two groups (p=0.363). A comparison with respect to lung involvement levels showed that the MMP-9/TIMP-1 ratio difference was found between the groups (Table 2).

The LDH, troponin, CRP, PCT, D-dimer, and ferritin values were found to be higher in the group with lung involvement than in the group without lung involvement. There was no difference between these two groups with respect to leukocyte, lymphocyte, and platelet counts (Table 3).

**Table 3:** Comparison of biochemical parameters of patients with and without lung involvement on chest CT scan

	Had no lung involvement on chest CT scan. (n=49)	Had lung involvement on chest CT scan. (n=143)	p
**Leukocyte count**	6,1 (2,4–14,0)	6,21 (2,7–505,0)	0,797
**Lymphocyte count**	1,4 (0,3–5,0)	1,2 (0,2–5,8)	0,857
**Platelet count**	191,0 (105,0–384,0)	211,5 (74,0–581,0)	0,799
**LDH**	196,0 (132,0–544,0)	312,0 (123,0–2433,0)	**0,001**
**Troponin**	2,1 (2,3–8,9)	4,3 (2,3–9722,0)	**0,001**
**PCT**	0,05 (0,05–0,15)	0,14 (0,05–10,0)	**0,002**
**Ferritin**	57,0 (3,6–268,6)	195,4 (3,4–1544,0)	**0,001**
**D-dimer**	254,0 (98,0–925,0)	847,0 (137,0–24100,0)	**0,001**
**CRP**	3,2 (1,0–74,0)	24,6 (1,0–425,0)	**0,001**

When the biochemical parameters were compared to the lung involvement levels, it was observed that as the level of lung involvement increased, the lymphocyte count decreased and the LDH, ferritin, PCT, CRP, D-dimer, and troponin values increased (Table 4).

**Table 4:** Comparison of biochemical parameters with respect to level of lung involvement on chest CT scan

	Group 2A (n=111)	Group 2B (n=16)	Group 2C (n=17)	p
**Leukocyte** **count**	6,1(2,4–22,0)	5,7 (3,6–505)	6,2 (2,7–21,7)	0,855
**Lymphocyte** **count**	1,4 (0,2–5,8)	0,9 (0,5–3,7)	0,7 (0,2–2,6)	**0,001**
**Platelet count**	213 (87–581)	186 (109–395)	143 (74–549)	0,078
**LDH**	221,5 (123,0–2433,0)	342 (229–486)	412 (221–645)	**0,001**
**Troponin**	2,4 (2,3–977,0)	5,1 (2,3–9722,0)	8,7 (2,3–48,0)	**0,001**
**PCT**	0,05 (0,05–10,0)	0,11 (0,05–0,30)	0,73 (0,05–2,8)	**0,001**
**Ferritin**	67 (3,4–1274,0)	312,1 (7,0–1544,0)	425 (21,0–1500,0)	**0,001**
**D-dimer**	298,5 (98,0–4330,0)	734,0 (184,0–5600,0)	964,0 (238,0–24100,0)	**0,001**
**CRP**	6,9 (1,0–425,0)	44,5 (4,2–112,0)	60,0 (4,4–329,0)	**0,001**

When the co rrelations between MMP-9, TIMP-1, and biochemical parameters were reviewed, MMP-9 was found to have a moderate positive correlation with the leukocyte count and a weak positive correlation with LDH, troponin, PCT, and CRP. TIMP-1 showed a weak positive correlation with ferritin and CRP (Table 5).

**Table 5:** Correlations of MMP-9 and TIMP-1 with biochemical parameters

		Leukocyte count	Lymphocyte count	LDH	Troponin	PCT	Ferritin	D-dimer	CRP
**MMP-9**	r p	0,441 **0,001**	-0,088 0,225	0,189 **0,010**	0,144 **0,046**	0,178 **0,013**	0,141 0,051	0,140 0,053	0,278 **0,001**
**TIMP-1**	r p	-0,050 0,490	-0,054 0,458	0,087 0,237	0,086 0,235	0,055 0,448	0,223 **0,002**	0,084 0,249	0,175 **0,015**

## Discussion

There is no COVID-19 study related to TIMP-1 in the literature. Our study is the first to investigate the relationship between COVID-19 and MMP-9 and TIMP-1.

A literature search revealed only a single study, by Ueland et al9., on MMP-9 in COVID-19. It included 39 adult patients. Plasma samples were collected three times: first in days 0–2, second in days 3–5, and third in days 7–10. Respiratory failure developed in 21 of the 39 COVID-19 patients who were being treated in the intensive care unit. The MMP-9 level was found to be higher in all three plasma samples (days 0–2, days 3–5, and days 7–10) of the patients who developed respiratory failure compared to those of the patients who did not develop respiratory failure. Assessing the course of MMP-9 values over time against respiratory failure showed that significantly high values of MMP-9 in the first two samples (days 0–2 and 3–5) could be an early indicator of respiratory failure in COVID-19 patients. This was explained by the fact that in ALI, MMP-9 secreted from neutrophils induces inflammation and degradation of the alveolar-capillary barrier, thereby further stimulating the migration of inflammatory cells and destruction of lung tissue.

The MMP-9 values of patients were found to be higher than those of the control group in our study. The MMP-9 were detected more in patients with lung involvement on chest CT scans than in those with no lung involvement on chest CT scans (p=0.023, p=0.035). This was linked to the fact that COVID-19-related pneumonia is characterized by increased fibrosis, reshaping of the extracellular matrix, and increased expression of MMP-9 secondary to inflammation in the lungs.

Ueland et al^9^. reported that male gender, high leukocyte and neutrophil counts, low lymphocyte counts, and high CRP and ferritin levels were poor prognostic factors. They also underlined that MMP-9 had a strong correlation with neutrophil counts and stressed that the new treatment methods targeting MMP-9 activity or neutrophil activation could be important options in the treatment of COVID-19-associated lung disease. Regarding the correlation of the biochemical parameters with the lung involvement levels on chest CT scans in our study, it was observed that with increasing lung involvement, the lymphocyte count declined and the LDH, ferritin, procalcitonin, CRP, D-dimer, and troponin values increased. MMP-9 was found to have a moderate positive correlation with leukocyte count and a weak positive correlation with LDH, troponin, PCT, and CRP. This suggested that MMP-9 was linked to the process of reshaping the damaged tissue in the lungs by way of inflammation, macrophage activation, and vascular inflammation.

A balanced MMP/TIMP ratio is important for lung tissue damage and repair6. In a prospective study investigating the MMP-9/TIMP-1 ratio in the pathogenesis of ARDS, 21 patients were divided into three groups: patients whose ARDS lasted less than 4 days (Group 1), patients whose ARDS lasted more than 8 days (Group 2), and a control group of patients diagnosed with hospital-acquired pneumonia in the absence of ARDS (Group 3). MMP-9, TIMP-1, and their ratios to each other were studied in BAL samples of patients at days 0, 4, 8, and 12. It was thought that the MMP-9/TIMP-1 ratio could indicate whether pulmonary fibrosis would be involved in ARDS or not. The study predicted that an MMP-9/TIMP-1 ratio of more than one (in other words, a higher level of MMP-9) could prevent the development of fibrosis by eliminating collagen and newly synthesized basal membranes in the lung tissue. It was concluded that a lower MMP-9 level (an MMP-9/TIMP-1 ratio less than one) could not prevent early fibroproliferation in ARDS. Interestingly, this ratio remained less than one in Group 2 with ongoing ARDS, but was more than one in the rapidly recovering group. These results show that the MMP-9 level and MMP-9/TIMP-1 ratio can be presented as predictive factors for the development of fibrosis in the lungs in conclusion, this study by Lanchou et al^8^. suggests that in the pathogenesis of ARDS, MMP-9 prevents the development of fibrosis in the lungs of patients by degrading extracellular matrix components that are synthesized by fibroblasts and plays an ant fibroproliferative role. The authors stated that the imbalance between MMP-9 and TIMP-1 can determine ARDS development and the tendency to pulmonary fibrosis.

Higher TIMP-1 values were found in the patients compared to the control group, and the rising levels of this molecule with increasing lung involvement in our study was linked to MMP-9 being a specific tissue inhibitor. Limitations of the study has got a small number of severely ill patients.

Therefore, there is a need for further molecular studies on the pathogenesis of COVID-19 and cytokine storms. Diagnostic and treatment methods targeting MMP-9 activity or neutrophil activation may be important in predicting lung involvement in COVID-19 and directing clinical outcomes.

## Information

This article was presented as an oral presentation at the 31st ECCMID congress held in Basel between 09-12 July 2021. (Summary number: 01155)
